# Development of Antibacterial Biocomposites Based on Poly(lactic acid) with Spice Essential Oil (*Pimpinella anisum*) for Food Applications

**DOI:** 10.3390/polym13213791

**Published:** 2021-11-01

**Authors:** Negin Noori, Ali Khanjari, Mohammadreza Rezaeigolestani, Ioannis K. Karabagias, Sahar Mokhtari

**Affiliations:** 1 Department of Food Hygiene, Faculty of Veterinary Medicine, University of Tehran, Tehran 14155-6453, Iran; khanjari@ut.ac.ir (A.K.); sahar.mokhtari416@gmail.com (S.M.); 2 Department of Food Hygiene and Aquaculture, Faculty of Veterinary Medicine, Ferdowsi University of Mashhad, Mashhad 91779-4897, Iran; mr.rezaee@ferdowsi.um.ac.ir; 3 Department of Chemistry, Laboratory of Food Chemistry, University of Ioannina, 45110 Ioannina, Greece

**Keywords:** antibacterial packaging, poly-lactic acid, physico-mechanical properties, anise, food

## Abstract

Among the main biodegradable food packaging materials, poly-lactic acid (PLA) is a commercially successful polymer used notably in the food packaging industry. In this study, active PLA films containing different percentage of anise essential oil (AE) (0, 0.5, 1 and 1.5% *v*/*v*) were developed, and characterized by physical, mechanical and antibacterial analysis. Based on physical examinations, thermal stability of PLA/AE films was greater than the neat PLA film, and the minimum water vapor permeability (WVP) was recorded for PLA/0.5AE film (1.29 × 10^−11^ g/m s Pa), while maximum WVP was observed for PLA/1.5AE (2.09 × 10^−11^ g/m s Pa). Moreover, the lightness and yellowness of the composites were decreased by the addition of AE. For the PLA composites with 1.5% AE, the tensile strength decreased by 35% and the elongation break increased by 28.09%, comparing to the pure PLA. According to the antibacterial analysis, the minimum inhibitory concentrations of PLA/AE film were 5 to 100 mg/mL and the active composite could create visible inhibition zones of 14.2 to 19.2 mm. Furthermore, the films containing AE inhibited *L. monocytogenes* and *V. parahaemolyticus* in a concentration-dependent manner. The confirmation of the success of the incorporation of EOs into the PLA films was further evaluated using principal component analysis, where positive results were obtained. In this context, our findings suggest the significant potency of AE to be used as an antibacterial agent in active food packaging.

## 1. Introduction

In response to growing concerns about the production of million tons of non-biodegradable plastics worldwide, attentions have been directed towards the development of new eco-friendly materials, particularly in the food packaging industry. It is increasingly gaining interest to use biopolymers for food packaging, due to depletion of oil resources and the adverse environmental impacts of petroleum-based plastics [1,2]. In this regard, many biodegradable polymers have been developed and evaluated, which among those materials poly-lactic acid (PLA) is a commercially successful polymer used notably in the food packaging industry [3]. Briefly, PLA is a biodegradable and biocompatible polyester made from natural resources, which is classified as generally recognized as safe (GRAS) for food packaging applications by the United States Food and Drug Administration (FDA) [4]. Beside the aforementioned properties of PLA, its promising mechanical and barrier features along with its retention properties of various molecules, altogether have made PLA an interesting bio-based base polymer for active food packaging applications [5]. Due to the increasing desire for high-quality, fresh and safe food products, the popularity of active packaging, as a great strategy for extending foodstuff shelf life, is continuously growing. Between various active packaging systems, antimicrobial packaging is a system that efficiently impregnates the antimicrobial agent into the food packaging material to enhance the product shelf life via suppressing the growth of foodborne pathogens and spoilage microorganisms [6]. It has been shown that PLA could be a fine base polymer material for the purpose of production of antimicrobial food packaging films, particularly in combination with protein antimicrobials, such as nisin [7], lysozyme [8] and pediocin [9]. Recently, impregnation of vegetable compounds such as herbal essential oils (EOs) and extracts into food packaging materials including PLA polymer has been taken into consideration by researchers [10,11]. However, so far, well-known herbs, such as oregano [12], thyme [13] and sage [14], were mainly considered as a source of antibacterial compounds in the research, while the potential application of other plants was less investigated. *Pimpinella anisum* (or anise) is one of those herbs that data are lacking about its capability to be used in the food industry. This medicinal plant belongs to the Apiaceae family, and the biological effects (i.e., antibacterial) of its EO are mainly attributed to the presence of active constituents such as volatile phenylpropanoids (i.e., anethole), estragole and anisaldehyde [15,16]. The flavoring feature of anise alongside its biological properties has made it an interesting option for its use as an additive in the food industry.

Considering the above, the aims of the present study were (i) to evaluate the antibacterial activity PLA films incorporated with anise essential oil (AE) against four food-related bacterial strains with disk diffusion method and (ii) to investigate physical and mechanical properties of the films.

## 2. Materials and Methods

### 2.1. Plant Material and Extraction of EO

*Pimpinella anisum* seeds (anise seeds, aniseed) were provided by Pakan Bazr Co. (Isfahan, Iran) and identified by an expert botanist at the department of food hygiene, Faculty of veterinary medicine, University of Tehran. Extraction of AE was carried out using the water distillation method described by Sharififar et al. [17]. In brief, 100 g of the seeds were submitted to water distillation in a clevenger apparatus for 3 h. The obtained oils were dried over anhydrous Na_2_SO_4_, and stored in the dark at 4 °C.

### 2.2. Chemical Analysis of EO

Chemical analysis of the resulting EO was carried out using a GC-MS system (Agilent 7890A/5975C), equipped with a capillary column (length: 30 m; diameter: 0.25 mm; film thickness: 0.25 μm). The temperature of the oven was programmed from 50 °C (3 min) to 240 °C (1 min) at a rate of 5 °C per min. The temperature of the injector and helium (carrier gas) flow rate were set at 250 °C and 1 mL per min respectively. The identification of EO constituents was done via comparison of the resultant GC-MS peaks with the Wiley (2001 data software), National Institute of Standards and Technology (NIST 08) commercial libraries and data from the literature.

### 2.3. Development of PLA Films

The active PLA films were prepared according to the method described by Abdulkhani et al. [18] by means of solvent casting technique with some modifications. PLA granules with a molecular weight of 197,000 g/mol and a density of 1.3 g/cm^3^ were supplied by FkuR kunststoffm (GmbH, Frankfurt, Germany). In brief, the granules were completely dissolved in a solvent (chloroform, Merck, Germany) by stirring with a magnetic bar for 8 h to achieve a PLA film-forming solution of 1% (*w*/*v*). For the preparation of PLA films, certain percentages of the prepared AE (0, 0.5, 1 and 1.5% *v*/*v*) were added to the PLA primary solution, and then the whole mixture homogenized (12,000 rpm for 2 min) using a homogenizer (T25, IKA, Staufen, Germany). All of the mentioned concentrations were chosen in accordance with the preliminary study and the literature [19]. Afterward, 50 mL of each solution was poured into 100-mm glass Petri-dishes, and the plates were placed under a chemical hood for 24 h at 25 °C to allow the solvent to evaporate. Next, the four types of PLA films (neat PLA and active PLA/AE composites) were aseptically peeled from the Petri-dishes and stored inside a desiccator containing silica gel until the time of antibacterial assay. Some typical PLA films (control) fabricated with *Pimpinella anisum* essential oil of different concentrations (0.5, 1, and 1.5%) are shown in Supplementary Figure S1.

### 2.4. Physical Characterization

#### 2.4.1. Thickness and Color Analysis

The color of the fabricated films was evaluated by measuring L* (lightness), a* (redness) and b* (yellowness) by means of a colorimeter (CR 300, Minolta Camera Co., Ltd., Osaka, Japan). For calibration of the colorimeter, a standard white plate was used. The determination of total color difference (ΔE) was carried out by using the following equation:

ΔE = ((ΔL*)^2^ + (Δa*)^2^ + (Δb*)^2^)^1/2^

where ΔL*, Δa* and Δb* are the difference of L*, a* and b* values of the PLA/AE composites with the same values of the neat PLA film, respectively.

The thicknesses of the composites were measured using a digital micrometer and an average of 10 measurements were recorded.

#### 2.4.2. Fourier Transform Infrared Spectroscopy (FTIR)

FTIR analyses of the PLA films were carried out using an ALPHA FTIR spectrometer (Bruker Optics Inc., Billerica, MA, USA). The spectra of PLA/AE bio-composite films were recorded from 4000 to 400 cm^−1^.

#### 2.4.3. Thermogravimetric Analysis (TGA)

TGA4000 thermogravimetric analyzer (Perkin-Elmer, Waltham, MA, USA) was used for analysis of thermal properties of the developed PLA films at a heating rate of 10 °C/min in the range of 30 to 600 °C under nitrogen atmosphere (20 mL/min).

#### 2.4.4. Differential Scanning Calorimetry (DSC)

DSC tests were carried out using a DSC4000 instrument in a nitrogen environment in the range of 30 to 600 °C. The heating and cooling rates were 10 °C/min (from 30 to 200 °C) and 100 °C/min (from 200 to 30 °C) respectively.

#### 2.4.5. Water Vapor Permeability (WVP)

WVP of the PLA films was measured gravimetrically according to ASTM E96 gravimetric method (2005). The films were cut into 4 cm circles and fastened on top of the circular glass cups containing six ml distilled water (100% RH; 2.337 × 10^3^ Pa water vapor pressure), and placed in a desiccator containing silica gel at 20 °C (0 Pa water vapor pressure). The quantity of weight loss from the glass cups was measured every 1 h during the first 9 h and every day during 4 days. WVP of the developed films were calculated using the following equation:

WVP = Δm X/A Δt Δp

where Δm/Δt is the weight gain or loss of glass cups as a function of time (g/s), X is the mean film thickness (mm), A is the surface area of PLA film (m^2^) and Δp is the partial water vapor pressure difference between two sides of the film (Pa).

### 2.5. Mechanical Properties

The mechanical characteristics of the active PLA films including tensile strength (TS), elastic modulus (EM) and elongation at break (EB) were evaluated by means of a Testometric Machine (M350-10CT model, Testometric Co. Ltd., Rochdale, Lancashire, UK) according to ISO 527-3. Prior to testing, all of the PLA films were equilibrated at 50% RH in a chamber containing magnesium nitrate saturated solution at 20 °C. The primary grip separation and crosshead speed were set at 50 mm and 50 mm/min, respectively. TS (MPa) was calculated by dividing the maximum load on the PLA film before failure by the cross-sectional area of the primary sample, and EM (GPa) was calculated according to the slope of the stress–strain curve in the linear range. EB (%) was defined as the percentage variation in the length of the film sample compared to the primary length between the grips.

### 2.6. Antibacterial Assay

#### 2.6.1. Bacterial Strains

In this study, four common foodborne pathogens were selected for antibacterial assessment of the films. Those bacterial strains included two Gram-negative bacteria namely *Vibrio parahaemolyticus* (ATCC 43996) and *Escherichia coli* O157:H7 (ATCC 43895), and two Gram-positive bacteria, namely *Staphylococcus aureus* (ATCC 25923) and *Listeria monocytogenes* (ATCC 19111). The stock cultures were prepared in Brain Heart Infusion (BHI) agar medium at 37 °C overnight. To prepare bacterial inoculums, bacterial cells were transferred to culture tubes containing BHI broth, incubated for 18 h at 35 °C and the cell density was adjusted to approximately 1 × 10^8^ CFU/mL by means of a spectrophotometer (Beckman, DU 530, Fullerton, CA, USA) at 600 nm. The BHI medium for *Vibrio parahaemolyticus* contained 1.5% NaCl and the incubation time was 6 h. BHI agar medium was employed for the estimation of the number of bacterial cells in the prepared inoculums.

#### 2.6.2. Antibacterial Properties of Films

For the evaluation of the antibacterial properties of the films, firstly, the minimum inhibitory concentration (MIC) of the developed composites was assessed against the abovementioned pathogens following the procedure detailed by Biswal et al. [20], and then the standard agar disc diffusion method was applied according to the method described in the literature [21].

For the determination of the MIC values of the films, different concentrations of film particles (3 mm × 3 mm) ranging from 1 to 100 mg in 1 mL of BHI broth solution were kept in a shaker incubator at 200 rpm for 24 h at 37 °C and then 1 mL of the supernatant was taken out from each concentration. Ten μL of the prepared pathogens cultures (having bacterial concentration of approximately 1 × 10^8^ CFU/mL) was inoculated into the extracted samples and incubated overnight at 37 °C. Fifty μL of the incubated solutions was transferred onto the previously prepared BHI agar plates and incubated for 24 h at 37 °C. The particle concentration required to completely inhibit the growth of the pathogens was estimated and reported as the MICs of the films.

To perform disc diffusion method, 100 μL of each bacterial suspension (containing ~1 × 10^7^ CFU/mL) were inoculated on Muller Hinton (MH) agar and incubated at 37 °C for 24 h. Then, the resultant PLA films were ascetically cut into a 9-mm circular shape using a hole puncher and placed on the surface of the inoculated-MH agar plates. After 24 h incubation at 37 °C, inhibition zones created surround the films discs were measured using a caliper and noted. The neat PLA film without the AE served as the control sample in both antibacterial tests.

### 2.7. Statistical Analysis

The physical, mechanical and antibacterial experiments were carried out in triplicate (except for thickness measurement with 10 replicates), and all data were reported as mean values with their standard deviation (SD) indicated. SPSS 16.0 software (Chicago, IL, USA) was employed for statistical analysis, and one way analysis of variance (ANOVA) by the Tukey’s test were performed to find the significant difference between the data recorded for the films. In this study, the statistical significance level was considered *p* < 0.05. To confirm the success of the incorporation of EOs into the polymer (PLA) principal component analysis (PCA) was carried out on the FTIR spectra using the Statgraphics Centurion XVI (version 16.1.15, Statgraphics Technologies, Inc., The Plains, VA, USA) statistical package.

## 3. Results and Discussion

### 3.1. Chemical Composition of EO

**Figure 1:**
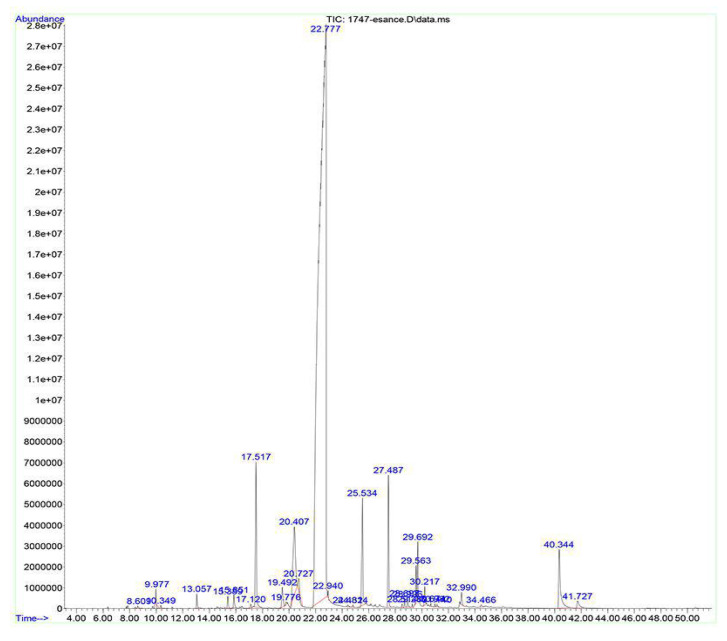
A typical total ion chromatogram (TIC) of *Pimpinella anisum* essential oil.

Totally 24 compounds were identified in the tested AE by means of GC-MS method (Figure 1). The main percentage of the oil belonged to the flavoring compound anethole (~85%), and the other main constituents were piperitenone oxide, p-allylanisole, trans-caryophyllene and acet-isoeugenol (Table 1). Likewise, the dominance of anethole in the EO of aromatic herbs such as anise, fennel and star-anise was reported in previous studies [15,22,23]. This monoterpene position isomer is used in pharmaceutical and food industries and the US Food and Drug Administration (FDA) has introduced anethole as a safe food flavoring additive [23].

Beside the non-toxic nature of anethole, significant bioactive properties, including antimicrobial, antiviral and antioxidative activity, have been reported for the compound in the literature [23]. The other minor elements presented in the AE such as caryophyllene and acet-isoeugenol are considered as flavoring agents for food application.

Generally, it is well demonstrated that the quantity and composition of herbal EOs are strongly influenced by climate conditions and the harvesting stage. In this regard, higher quality and better yield of AE have been connected to harvesting anise at the initiation of the waxy stage and production of the herb in warm climate conditions respectively [24].

### 3.2. PLA Film Characterization

#### 3.2.1. Thickness and Color Analysis

The thickness and color properties of the developed PLA films are presented in the Table 2. The incorporation of higher concentrations of AE (1 and 1.5% *v*/*v*) increased the thickness of the composites (*p* < 0.05), and PLA/1.5AE was ~11% thicker than the pure PLA film. However, the addition of 0.5% AE could not affect the thickness. The positive influence of the incorporation of EO into the casted PLA films was also previously stated [25].

The developed films were visually clean and transparent without any particulate area. The evaluation of color parameters of the composites revealed that with the addition of AE lightness and yellowness values were decreased while a* was elevated. In fact, PLA/1.5% with the highest ΔE value (4.51) was the reddest, bluest and darkest film simultaneously in comparison with the other PLA/AE films. This might be due to the coloring substances naturally presented in AE. In general, morphological properties and color profile of a film are governed by multiple parameters, including the color of base-polymer and additive material, interactions between the components and the method of manufacturing.

According to the previous studies, different herbal EOs had different impacts on the color properties of PLA films. For instance, Sharma et al. showed that with the addition of different concentrations of clove and thyme EO (1 wt%, 5 wt% and 10 wt% of polymer resin) b* and a* values of PLA/Poly (butylene adipate-co-terephthalate) films were increased and decreased respectively [26]. These results beside the values reported for PLA/clove or peppermint EOs [27] are contrary to our findings. Other researchers stated different patterns of color changes such as an increase of redness and yellowness by the addition of 1% (*v*/*v*) *Zataria multiflora* Bioss. EO into PLA film [28]. However, none of those changes nor ours were not drastic and mainly less than 10% variation recorded for the enhanced bioactive PLA films.

#### 3.2.2. FTIR Analysis and Confirmation of the Success of the Incorporation of EOs into the PLA

**Figure 2:**
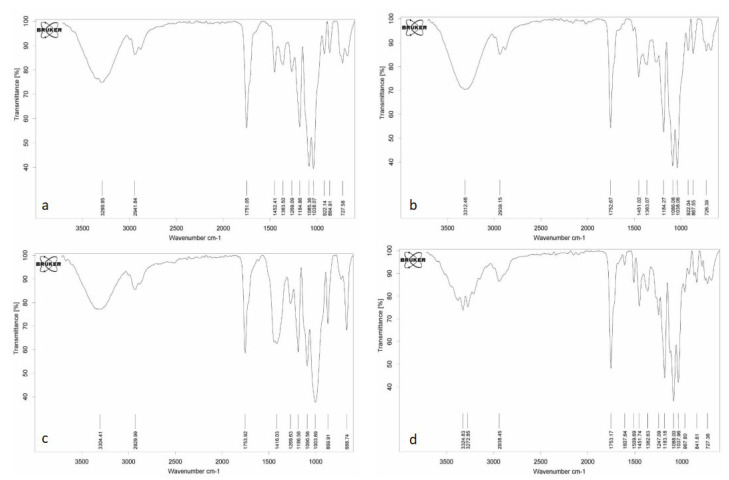
FTIR spectra of PLA films incorporated with different concentrations of anise essential oil (AE): neat PLA (**a**); PLA/0.5AE (**b**); PLA/1AE (**c**); PLA/1.5AE (**d**).

Figure 2a–d represent the FTIR spectrum of control PLA films and those fabricated with different concentration of anise essential oil (0.5, 1, and 1.5% *v*/*v*). Before going any further it is important to stress that FTIR is a spectroscopic technique that is widely used to find out the infrared (IR) spectrum of absorption or emission of the sample of interest. IR absorption spectra imitate absorption bands that are related to various available functional groups. In that sense, FTIR is a mandatory technique for the characterization of packaging films fortified with different materials. Indeed, there were observed differences between PLA films fabricated with different concentration of anise essential and the control sample. The frequency bands at 2941.84 and 2929.99 cm^−1^ indicate C–H stretching for alkanes. The peaks at 1753.17 to 1751.05 cm^−1^ reveal the presence of C=O bonds in carbonylic compounds (aldehydes). The frequency bands at 1363.50 and 1452.41 cm^−1^ in PLA films fabricated with anise essential oil indicate the C-H alkane scissoring and bending [3]. Bands obtained between 922.04 cm-1wavelength and 967.80 cm^−1^ wavelength reveal the existence of functional groups such as –C–O and–C=O which are probably related to the terpenoids of the anise essential oil. In the same line of reasoning, bands obtained between 675–1000 cm^−1^ indicate the C-H alkene bend [1] which is probably related to terpenoids. The bands between 727.58 and 726.39 cm^−1^ indicate the presence of aromatic C=C [3] in PLA films fabricated with anise essential oil, whereas no band in this wavelength was monitored for control PLA films. For the PLA films fabricated with the higher concentration of anise essential oil (1.5% *v*/*v*) the shift of band at 1607.74 cm^−1^ may indicate the existence of fatty acids in the PLA films. Finally, the frequency bands at 922.14 and 922.04 cm^−1^ indicate the -CH=CH_2_ stretching [2]. Present results are in agreement with those reported by Anuar et al. (2017) for PLA films fabricated with cinnamon essential oil [29].

**Figure 3:**
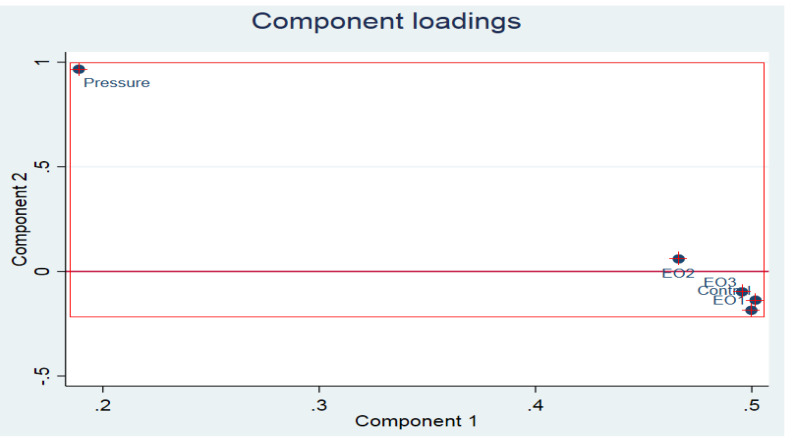
Plotting of the observations related to the FTIR spectral data of the variables (control: PLA films, EO1: PLA films incorporated with 0.5% of anise EO (PLA/0.5AE), EO2: PLA films incorporated with 1% of anise EO (PLA/1AE), EO3: PLA films incorporated with 1.5% of anise EO (PLA/1.5AE)) loaded on the multidimensional space of PCA as principal components 1 and 2 (PC1 and PC2). In particular, 0.2 or 20% of the pressure (indication of the successive passing of the tolerance test) on PC 1 and nearly 100% (≥97%) of the pressure on PC2 are loaded.

As mentioned before, the success of the confirmation of the incorporation of the EOs into the PLA was established using PCA on the FTIR spectral data. The Kaiser–Meyer–Olkin (KMO) measure which is a criterion of the sampling adequacy for PCA (should be ≥0.50) had the value of 0.8303 (Figure 3), indicating that PCA could be established on the FTIR spectral data of the biocomposites incorporated with different concentrations of *Pimpinella anisum* essential oil. The total variance explained was 97%. The correlation values of the control (PLA), and PLA films fabricated with 0.5%, 1% and 1.5% were 0.2483, 0.9913, 0.8460 and 0.9732, respectively. The differences in the correlation values indicate the successful incorporation of the EOs into the PLA films.

#### 3.2.3. TGA

**Figure 4:**
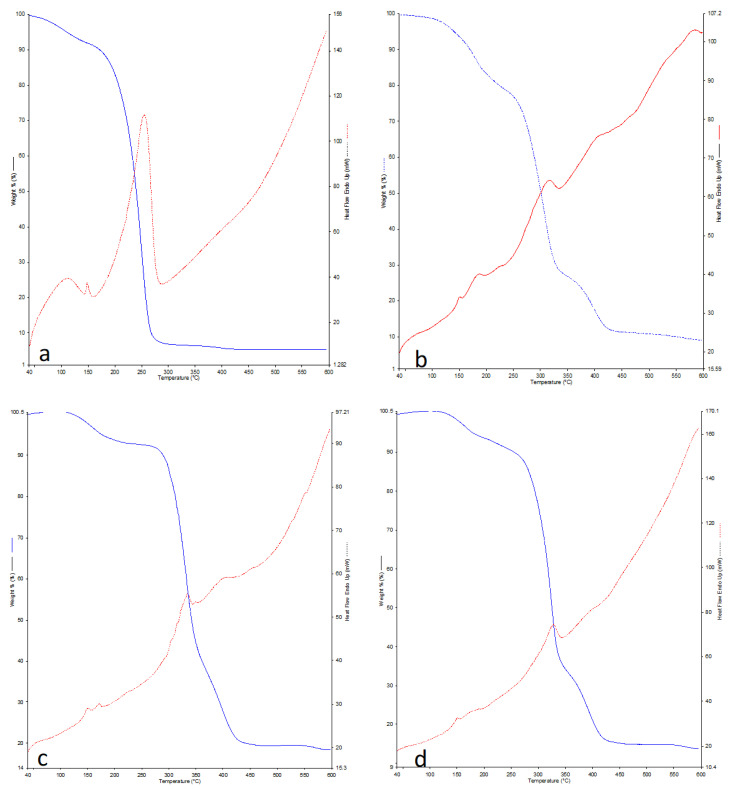
Thermal analysis of PLA films incorporated with different concentrations of anise essential oil (AE): neat PLA (**a**); PLA/0.5AE (**b**); PLA/1AE (**c**); PLA/1.5AE (**d**).

The TGA analysis was performed to study the influence of the AE on thermal stability of PLA-based composites. The TGA curves of neat PLA and PLA/AE composites are shown in Figure 4a–d. As shown in Figure 4 the thermal weight loss of PLA films was entirely sensitive to temperature and the decomposition of PLA was occurred in a single-step degradation process.

The onset of degradation temperature (T_onset_), the maximum degradation temperature (T_max_: the temperature at the maximum weight-loss rate), which determined from the maximum temperature of the peak in TGA curve derivatives, and thermal stability of PLA increased with the incorporation of different concentrations of AE in PLA matrix. Moreover, the addition of oil into the polymer matrix led to a two-step decomposition process, where the initial degradations step was more pronounced for PLA/1AE and PLA/1.5AE films. This step was most probably corresponding to the AE degradation, while the second decomposition step was due to the decomposition of PLA.

Changes in thermogravimetric curves and increase in thermal stability have also been previously reported for PLA/EO composites [29,30]. Qin et al. [30] stated that the incorporation of EOs of different herbs (like bergamot, lemongrass, rosemary, and clove) created another degradation step in the thermogravimetric analysis of PLA-based films. They have attributed the small wight loss during the first decomposition phase to gradual evaporation of EO moisture. Regarding the improvement in the thermal stability of PLA/EO composites, Anuar et al. [29] reported that the PLA biocomposite containing 1wt% cinnamon EO (CEO) and palm oil could better withstand the thermal stress compared to neat PLA. The formation of hydrogen bonds between the chains of PLA and palm oil was mentioned as the probable factor enhancing the thermal stability of the resultant film. In this regard, similar phenomenon might be occurred in the matrix of PLA/AE composites where the formation of hydrogen bonds between the PLA and AE might delay the degradation of the films.

#### 3.2.4. DSC

The effect of AE on the melting behavior and crystallization of the PLA-based films was studied in DSC experiments. The DSC thermograms of neat PLA and PLA/AE composites are shown in Figure 4. Some important parameters like melting temperature (Tm) and decomposition temperature (Td) of the fabricated films were estimated from the DSC curves. Pure PLA film exhibited two distinct peaks at 158 °C and 265 °C corresponded to T_m_ and decomposition temperature of PLA. These results agree with the results demonstrated previously for PLA [31,32]. With the addition of different percentages of AE, the extent of the aforementioned peaks was decreased and the T_m_ of the resultant composites remained almost unchanged. Yahyaoui et al. [33] also showed that no significant changes occurred in melt temperature of PLA films incorporated with EOs from rosemary, myrtle and thyme. Unlike Tm, the decomposition temperature of PLA/AE films increased to 314–333 °C. This finding is in good agreement with the TGA results, where the incorporation of AE into PLA matrix delayed the decomposition of the composites. It seems that the hydrogen bonds between PLA chains and AE components play an important role in increasing the thermal stability of the films.

#### 3.2.5. Water Vapor Permeability

**Figure 5:**
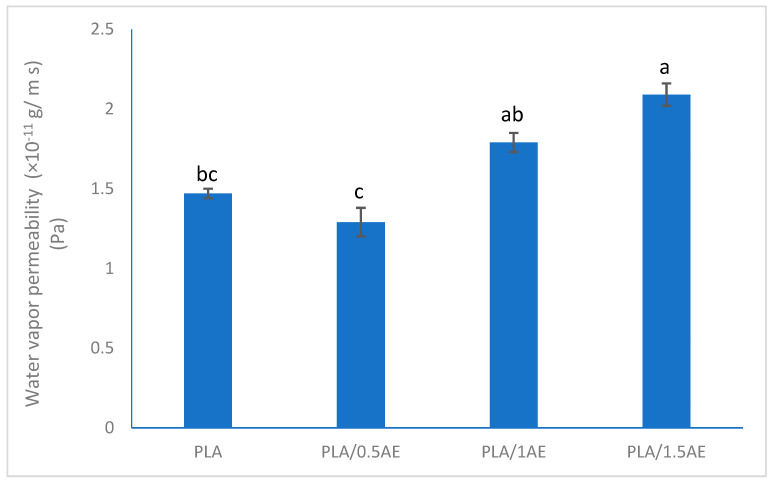
Water vapor permeability of poly lactic acid (PLA) films incorporated with anise essential oil (AE). Different lowercase letters indicate significant differences (*p* < 0.05).

One of the main physical properties of a packaging film is its water vapor barrier performance which plays an important role, particularly in fresh food packaging. In general, PLA is more permeable to water vapor comparing to petroleum-based packaging materials like polyethylene [34]. The WVP of the pure PLA was comparable to the values reported for similar casted PLA films [28]. Figure 5 shows the impact of different percentages of AE on water barrier properties of PLA films. The minimum WVP was recorded for PLA/0.5AE film while higher concentrations of AE made the PLA films more water vapor permeable. Although the WVP of PLA/0.5AE was lower than the pure film, this difference was not significant (*p* < 0.05).

The impacts of herbal EOs and their main constituents on WVP of various packaging films including PLA have been previously evaluated. In this regard, main reports indicated the negative influences of different types of EO (or their constituents) on the barrier performance of the composites.

For instance, WVPs of Poly (Lactic A id)/Poly(Trimethylene Carbonate) and PLA films were reduced by adding oregano and thyme EO, respectively [19,28]. The same changes were also observed for PLA composites enhanced by thymol [35] or limonene [36]. Several reasons have been proposed for this phenomenon, including the increase of the average pore size of the films, plasticizing effects of the oil and weaker structure of PLA/oil composites [21,36].

However, the lower water barrier performance of a food packaging film does not necessarily mean that that film is not appropriate for food application. Rather, this feature would be beneficial for post-harvest fruit and vegetable quality by reducing the risk of condensation within the package [37]. Moreover, the developed PLA/AE films had adequate barrier properties to be used for other types of food products.

### 3.3. Mechanical Properties

One of the main aspects of the characterization of newly developed materials is the mechanical properties of the composite and for this purpose different parameters like TS, EM and EB are normally characterized. Table 3 shows that TS and EM values of the composites were declined with the addition of the EO in a concentration-dependent manner, while EB percentages were increased (*p* < 0.05).

The main reports existed in the literature concerning the mechanical evaluation of PLA/EO films confirm our findings. In a study, active PLA-essential oil composites were developed by the addition of different concentrations of oregano EO (GE) (0.5, 1 and 1.5% (*w*/*w*)) for food application [25]. While both TS and EM were decreased, a remarkable increase in EB was reported for the PLA/GE films (up to 6-fold comparing to neat PLA. Regarding the lower content of the used EO in PLA/GE films in comparison with the AE applied in the present work, the degree of the EB reduction was far higher for the PLA/oregano oil films. However, the EB values of PLA/GE (2.82–16.78%) were significantly lower than the values recorded in this study showing the higher flexibility of PLA/AE films. Our EB data was closer to the values reported by Rezaeigolestani et al. [28] for PLA/*Zataria multiflora* Bioss. films with EB% of 39.6–74.6. The plasticizing effect of EOs was mentioned as the most probable cause of higher flexibility of PLA/EO composites through facilitating the movement of polymer chains [38]. Therefore, since one of the main shortcomings of PLA for food applications is its brittleness, AE could efficiently overcome this flaw and provide a more flexible film for food packaging.

The decline in TS of PLA films has also been reported by the addition of clove and oregano oil [19,26]. It seems that higher concentrations of EO resulted in a phase separation within the structure of active films and consequently the mechanical integrity of the composites was dropped.

### 3.4. Antibacterial Properties of Films

In the modern food industry, the application of active compounds in order to extend shelf life of perishable food products has gained important attention. Foodborne bacterial pathogens and also spoilage bacteria are among the most important concerns threatening the safety and quality of foodstuffs. Therefore, the development and application of active films or coatings containing antibacterial compounds have taken priority over other types of active food packaging materials.

**Figure 6:**
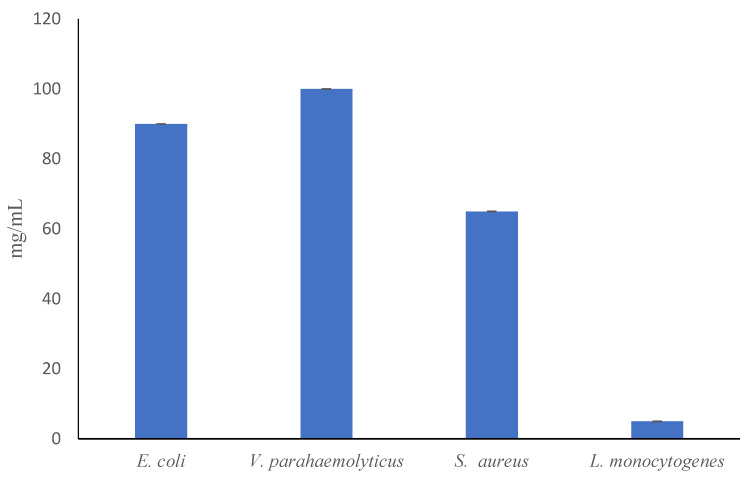
Minimum inhibitory concentration (MIC) of poly lactic acid (PLA) film containing anise essential oil against four food-borne pathogens.

The minimum concentration of the PLA/AE film particles that could entirely inhibit the growth of the tested pathogens are shown in Figure 6. The pure PLA film as control could not affect the growth of the tested strains and the MIC values for PLA/AE particles ranged between 5 to 100 mg/mL. As it can be seen in Figure 6, *L. monocytogenes* and *S. aureus* were more sensitive toward the film particles comparing to *E. coli* and *V. parahaemolyticus.* These findings are in good agreements with the disc diffusion results and the data reported by Khanjari et al. for anise *(Pimpinella anisum)* EO [39]. The MICs of pure AE reported in the previous work by Khanjari et al. [39] against the similar strains were between 150 and 1200 μg/mL, and *L. monocytogenes* and *V. parahaemolyticus* were similarly the most and least vulnerable strains, respectively. As expected, the MICs recorded for the PLA/AE film in present study were far higher than the reported MICs for pure AE in the aforementioned study. The similar observation was made by Biswal et al. [20], who demonstrated a considerable antibacterial activity of thymol released from PLA-based micro-particles.

The antibacterial activity of the fabricated PLA films against the tested foodborne pathogens evaluated by disc diffusion agar is shown in Table 4. As expected, the pure PLA film could not create any visible inhibition zone in the bacterial plates. The active films inhibited the growth of three pathogens (*E. coli*, *V. parahaemolyticus* and *S. aureus*) almost equally, which the relevant inhibition zones ranged between 14.2 mm and 19.20 mm. Besides, *Listeria monocytogenes* was the most sensitive strain to the antibacterial activity of the fabricated films. Although, the addition of different concentrations of AE had different outcomes, in most of the cases incorporation of higher percentages of the oil was more effective against the growth of the bacteria. Our results showed that while no definite relationship was recorded between the concentration of AE and the extent of *S. aureus* and *E. coli* growth inhibition zones, the films containing AE inhibited *L. monocytogenes and V. parahaemolyticus* in a concentration-dependent manner. As shown in Table 4, the antibacterial effects of gentamicin as control positive were comparable to the PLA/AE films developed in this study, and in the case of *L. monocytogenes*, PLA/1AE and PLA/1.5AE exhibited even greater inhibition zone comparing to gentamicin.

Based on the previous works on the evaluation of antibacterial properties of herbal EOs, in general, Gram-positive bacteria are more vulnerable to destructive effects of EOs due to the lack of lipopolysaccharide layer around the cell wall of these bacteria [28,42]. However, it seems that among the two tested Gram-positive pathogens only the growth of *L. monocytogenes* was more greatly inhibited by the active films (especially with 1% and 1.5% AE) in comparison to the other Gram-negative strains (*p* < 0.05).

In general, the agar disc diffusion method is always considered as one of the main approaches for evaluating bioactive properties of food packaging materials owing to its capability to be used as a quantitative assay and to simulate wrapping foodstuffs. There is a great burden of studies examined the antibacterial activity of original food packaging composites or coatings using disc diffusion method. They assessed different packaging polymers incorporated or coated with different active compounds including natural bio-active substances. However, among those polymer/active agent combinations, active PLA composite films impregnated with natural antimicrobials have been recently well evaluated [8].

The results of some of those studies on the fabrication of antibacterial PLA films are compared in Table 5. Different types of antibacterial compounds with different concentrations were employed for the production of active PLA composites, where solvent casting and melt blending were the most common methods used for the production of the films. The active agent content of the developed composites varied noticeably (0.5–80%), and hence the recorded bacterial inhibition zones were extremely diverse ranged between 2.33 mm to 80 mm. Therefore, in order to better compare the antibacterial efficacy of the composites, Antibacterial Effectiveness Factor (AEF) was defined as follow:

AEF = Inhibition zone diameter (mm)/active agent content in the composite (%)

In this regard, the active PLA/oregano EO films developed by Javidi et al. [24] possessed the best AEF (11.7 and 10.13 against *S. aureus*), while the least AEF was recorded for the PLA composite contained garlic EO (against *S. aureus*) [43].

A comprehensive literature review shows that main factors determining antibacterial potency of a composite film include the type of the polymer, antibacterial activity of the active agent, method of composite manufacturing, conditions of antibacterial testing, similarity between the nature of polymer and antibacterial (hydrophobicity or hydrophilicity) [8,28].

Higher temperature can improve the antibacterial activity of a composite by expanding the polymer matrix and providing wider paths for antibacterial release. This was indicated by several authors including Rezaeigolestani et al. [28] for PLA/*Zataria multiflora* EO and also it can be seen in the results of Ahmed et al. [43], where the antibacterial effects of the active PLA/garlic EO films were drastically higher in the case of *Campylobacter jejuni* tested at 42 °C.

Table 5 demonstrates that olive leaf extract, oregano EO and *Zataria multiflora* EO were more effective against the tested pathogens in comparison to the examined AE. Meanwhile, it seems that the antibacterial activity of AE in the matrix of PLA films overcomes over the relevant effects of garlic EO and lauric arginate.

Comparison of active compound content in the PLA films presented in Table 5 indicates that lack of antibacterial effect for some of those composites (i.e., PLA/Allium spp. extract composites) may be related to the lower percentage of the applied agent. Nevertheless, the prosperous incorporation of a lower proportion of oregano EO into PLA matrix (the highest AEF values in Table 5) [25] showed that significant antibacterial properties of an antibacterial can overcome the need for the addition of a greater percentage of the agent.

Several locations (i.e., cytoplasmic membrane) and some mechanisms (i.e., proton motive force (PMF), active transport and electron flow) in the bacterial cells are recognized as the action sites for herbal EOs constituents [42,44]. Based on the literature and chemical analysis of the tested AE, the presence of important antibacterial compounds such as anise camphor (anethole) and terpene hydrocarbons alongside the suppressing ability of anise EO to the adhesions of bacterial cells might explain the antibacterial impacts of the active PLA/AE films [22,45].

## 4. Techno-Economic Challenges of the Developed Biocomposites

Despite the fact that PLA is among the most popular and affordable biodegradable polymers, the cost of herbal EOs and the technological difficulties in developing these types of composite films are among the primary issues that must be considered in the future studies for a possible application in the food industry. At the same time, the use of the active agents of EOs and the implementation of other film making technologies (like film extrusion) might facilitate the commercialization of PLA/EOs composites and make their use more common.

## 5. Conclusions

This paper investigated the physical, mechanical and antibacterial performances of novel PLA-based composite films containing anise EO intended to be used in food packaging. Based on physical examinations, the color changes induced by AE were not drastic and the active PLA/AE films had acceptable water vapor barrier performance. Moreover, thermal stability of PLA increased with the incorporation of different concentrations of AE in PLA matrix. Mechanical analysis showed that the flexibility of the films was desirably increased by the addition of AE. Our findings revealed that the resultant active films could efficiently inhibit the growth of the tested bacteria, sometimes in concentration-dependent manner. Regarding the data of previous studies on antibacterial properties of active PLA films containing different active compounds, the bactericidal-bacteriostatic quality of AE was relatively remarkable, and this adds to the growing body of literature on the great potency of EOs to be used in food packaging.

## Figures and Tables

**Table 1:** The chemical composition of *Pimpinella anisum* essential oil.

Compounds	RT ^a^ (min)	GC Area (%) ^b^
Anethole	22.791	80.84
Piperitenone oxide	20.408	5.76
p-Allylanisole	17.517	2.9
Acet-isoeugenol	40.343	2.05
trans-Caryophyllene	27.49	2.05
Germacrene-D	29.693	0.76
Pulegone	19.489	0.52
1,8-Cineole	9.973	0.33
Camphene	30.217	0.29
Linalool	13. 06	0.23
Menthone	15.853	0.21
L-Menthone	15.391	0.2
trans-beta-Farnesene	28.928	0.14
alpha-Humulene	28.697	0.13
alpha-Terpineol	17.122	0.07
Spathulenol	34.463	0.06
trans-beta-Ocimene	10.348	0.05
3-Octanol	8.607	0.04
p-Allylanisole	24.434	0.04
Camphene	24.814	0.04
Benzene, 1-methyl-3-(1-methylethyl)-	9.737	0.03
2-beta-Pinene	7.847	0.03
alpha-Pinene	6.358	0.02
Sabinene	7.739	0.02

^a^ Retention time. ^b^ Relative proportions as percent of the total peak area.

**Table 2:** Physical properties of poly lactic acid (PLA) films incorporated with anise essential oil (AE) ^1^.

		Color Parameters			
Films	Thickness (mm)	L*	a*	b*	ΔE
PLA	0.078 ± 0.01 ^a^	83.11 ± 0.67 ^a^	0.75 ± 0.06 ^d^	15.23 ± 0.12 ^a^	0
PLA/0.5AE	0.079 ± 0.03 ^b^	82.26 ± 0.54 ^a^	1.06 ±0.03 ^c^	14.51 ± 0.09 ^ab^	1.15
PLA/1AE	0.084 ± 0.04 ^a^	80.92 ± 0.88 ^ab^	1.21 ± 0.03 ^b^	13.84 ± 0.15 ^bc^	2.63
PLA/1.5AE	0.087 ± 0.02 ^a^	79.15 ± 0.71 ^b^	1.37 ±0.04 ^a^	13.15 ± 0.18 ^c^	4.51

^1^ Values are expressed as mean ± standard deviation (n = 3). ^a–d^ Different lowercase letters within a column indicate significant differences (*p* < 0.05).

**Table 3:** Mechanical properties of poly lactic acid (PLA) films incorporated with anise essential oil (AE) ^1^.

Films	Tensile Strength (MPa)	Elastic Module (GPa)	Elongation at Break (%)
PLA	18.6 ± 0.73 ^a^	0.96 ± 0.12 ^a^	52.47 ± 1.35 ^d^
PLA/0.5AE	15.23 ± 0.52 ^b^	0.85 ± 0.08 ^a^	56.25 ± 0.75 ^c^
PLA/1AE	10.19 ± 0.47 ^c^	0.59 ± 0.13 ^b^	61.19 ± 2.21 ^b^
PLA/1.5AE	6.51 ± 0.24 ^d^	0.41 ± 0.05 ^c^	67.21 ± 1.44 ^a^

^1^ Values are expressed as mean ± standard deviation (n = 3). ^a–d^ Different lowercase letters within a column indicate significant differences (*p* < 0.05).

**Table 4:** Antibacterial evaluation of different poly lactic acid (PLA) films containing anise essential oil against four bacterial pathogens by disk diffusion method at 37 °C.

	Inhibition Zone Diameter (mm) *				
Films	*E. coli*	*V. parahaemolyticus*	*S. aureus*	*L. monocytogenes*	Ref.
PLA	_	_	_	_	Present study
PLA/0.5AE	14.2 ± 0.6 ^aA^	15.46 ± 0.11 ^aA^	14.90 ± 0.17 ^aA^	15.55 ± 0.05 ^aA^	Present study
PLA/1AE	14.17 ± 0.13 ^aA^	16.33 ± 0.35 ^aA^	15.13 ± 0.52 ^aA^	26.09 ± 1.79 ^bB^	Present study
PLA/1.5AE	15.19 ± 0.63 ^aA^	19.20 ± 0.63 ^bB^	14.5 ± 0.34 ^aA^	34.34 ± 4.01 ^cC^	Present study
GM (10 μg)	19.5	Not reported	25	22	[40,41]

* Values are expressed as mean ± standard deviation (n = 3). _: No inhibition zone detected around the discs. ^a–c^ Different lowercase letters within a column indicate significant differences (*p* < 0.05). ^A–C^ Different lowercase letters within a row indicate significant differences (*p* < 0.05). GM: gentamicin; positive control.

**Table 5:** Comparison of antibacterial effects of different active poly-lactic acid (PLA) films against common foodborne pathogens.

Film Composition	Method of Active Film Manufacturing	Inhibition Zone Diameter (mm)	Bacterial Strain	Antibacterial Effectiveness Factor (AEF)	Ref.
PLA/6.25 wt% olive leaf extract (OLE)	Solvent casting	9.1	*S. aureus*	1.456	[21]
PLA/18.75 wt% OLE		13.5		0.72	
PLA/37.5 wt% OLE		16.2		0.432	
PLA/0.5 wt% oregano EO	Solvent casting	3	*S. aureus*	6	[25]
		-	*E. coli*	-	
PLA/1 wt% oregano EO		11.7	*S. aureus*	11.7	
		3.7	*E. coli*	3.7	
PLA/1.5 wt% oregano EO		15.2	*S. aureus*	10.13	
		10.6	*E. coli*	7.06	
PLA-PEG ^1^/80 *v*/*w*% garlic EO	Solvent casting	5	*S. aureus*	0.06	[43]
PLA-PEG/80 *v*/*w*% garlic EO		45	*Campylobacter jejuni*	0.56	
PLA-PEG/80 *v*/*w*% clove EO		10	*S. aureus*	0.12	
PLA-PEG/80 *v*/*w*% clove EO		80 (complete)	*Campylobacter jejuni*	1	
PLA/2, 5, 6.5 wt% Allium spp. extract	Melt blending	-	*E. coli* O157:H7	-	[46]
		-	*S. aureus*	-	
PLA/8 wt% Ag	Solvent casting	4	*S. aureus*	0.5	[47]
		1.43	*E. coli*	0.17	
PLA/16 wt% Ag		8	*S. aureus*	0.5	
		2.33	*E. coli*	0.14	
PLA/32 wt% Ag		9.33	*S. aureus*	0.29	
		2.33	*E. coli*	0.07	
PLA-CNF ^2^/20 *v*/*w*% *Zataria multiflora* EO	Solvent casting	31	*S. aureus*	1.55	[28]
		28	*E. coli*	1.4	
PLA-CNF/33 *v*/*w*% *Zataria multiflora* EO		39	*S. aureus*	1.18	
		32	*E. coli*	0.96	
PLA/28 wt% lauric arginate (LAE)	Melt blending/coating	4	*L. monocytogenes*	0.14	[48]
		4	*S. Typhimurium*	0.14	

- Not detected. ^1^ Poly ethylene glycol. ^2^ Cellulose nanofiber.

## Supplementary Materials

The following are available online at https://www.mdpi.com/article/10.3390/polym13213791/s1, Figure S1. Typical PLA films (control) incorporated with *Pimpinella anisum* essential oil of different concentrations (0.5, 1, and 1.5%) as reported in the study.
