# Supplementary material for: Development of Antibacterial Biocomposites Based on Poly(lactic acid) with Spice Essential Oil (Pimpinella anisum) for Food Applications
Source: Polymers (Basel). 2021 Nov 1;13(21):3791. doi: 10.3390/polym13213791 (PMC8587201; doi:10.3390/polym13213791)
Supplement: Supplementary file 1 [file polymers-13-03791-s001.zip › polymers-1217444-supplementary.pdf]

polymers

MDPI

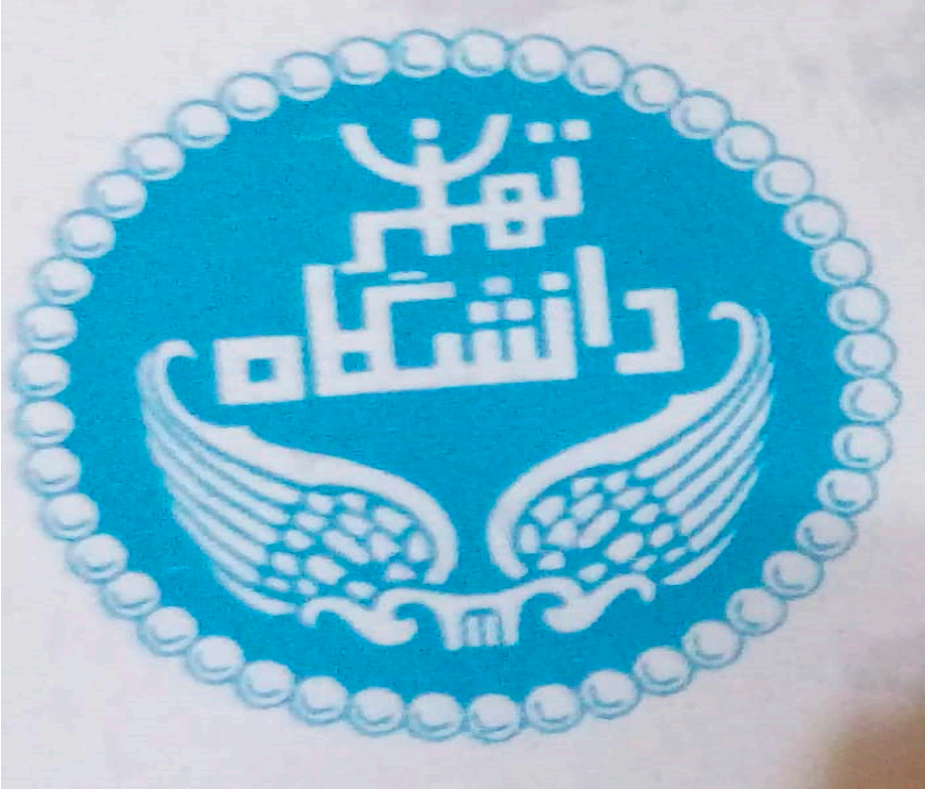

Logo without the films.

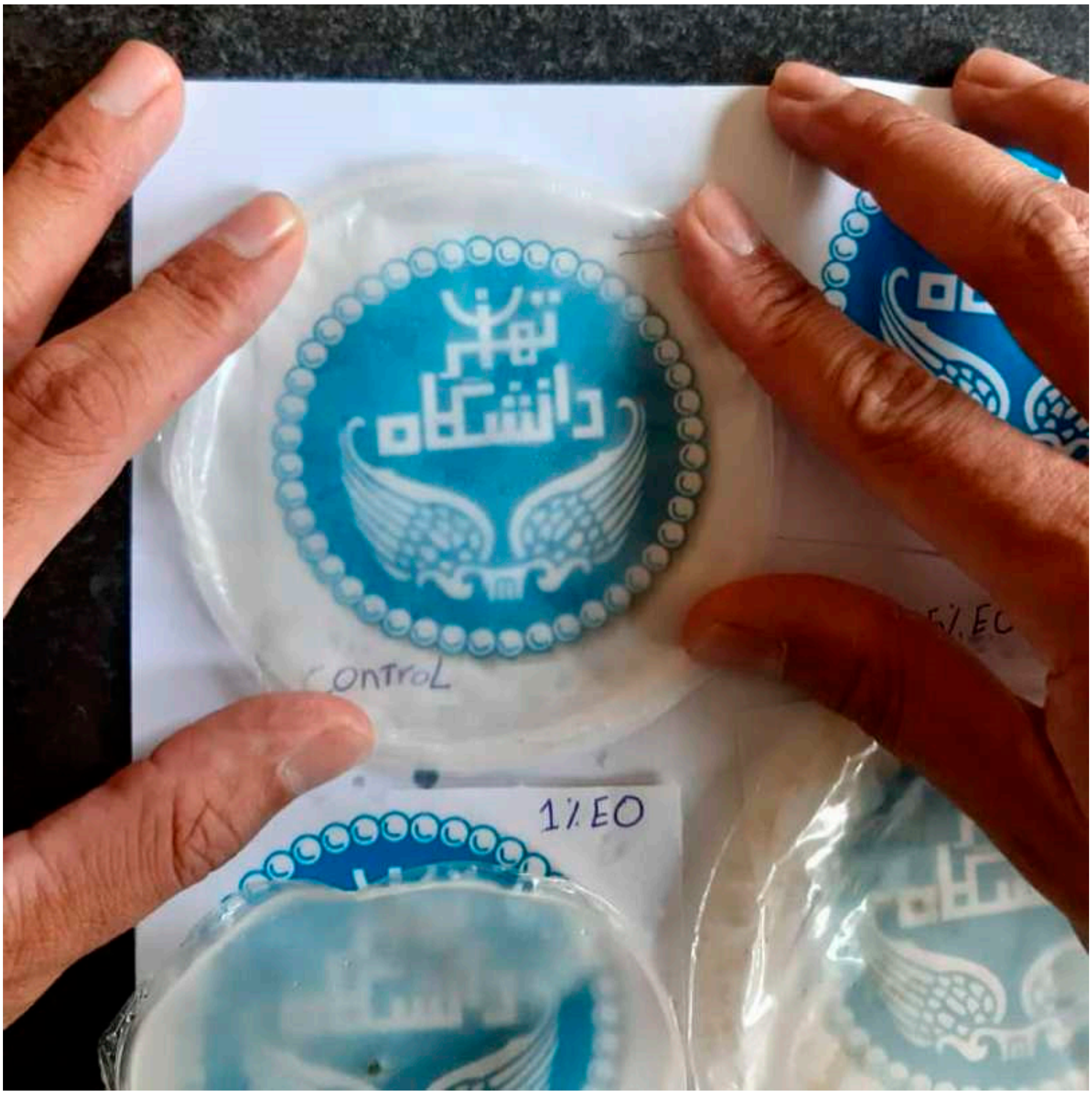

Figure S1. Typical PLA films (control) incorporated with *Pimpinella anisum* essential oil of different concentrations (0.5, 1, and 1.5%) as reported in the study.

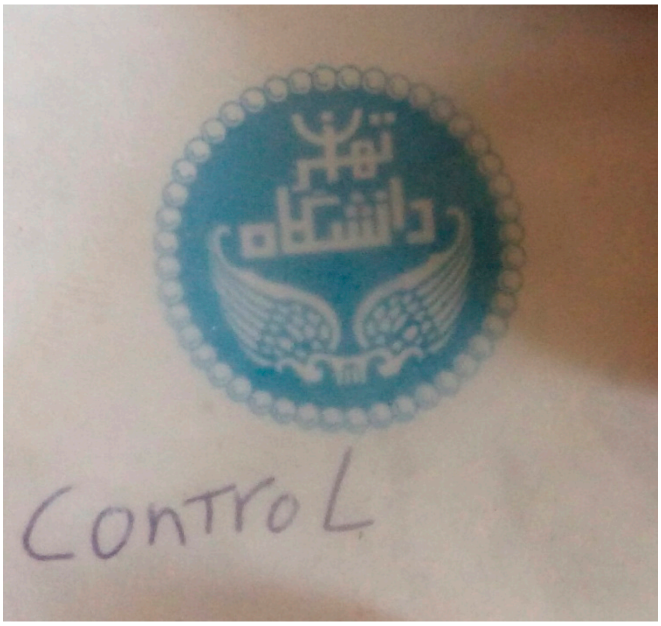

Control (PLA).

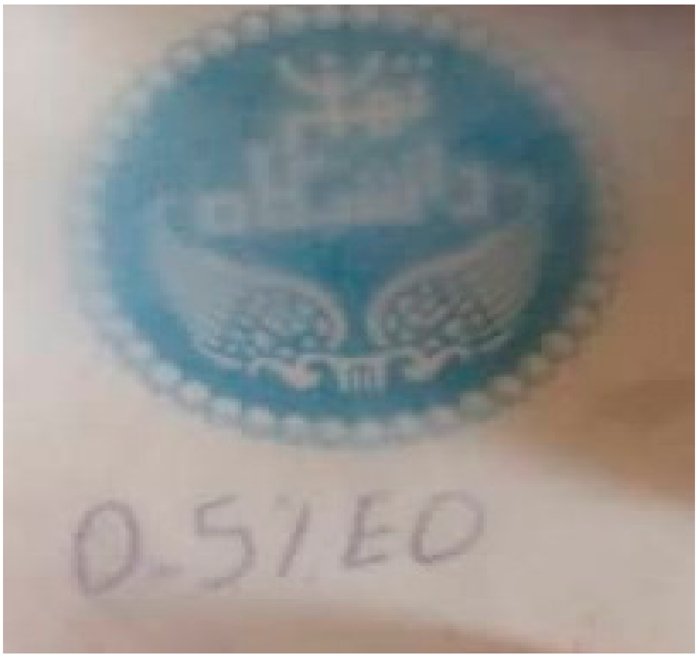

PLA+0.5% EO.

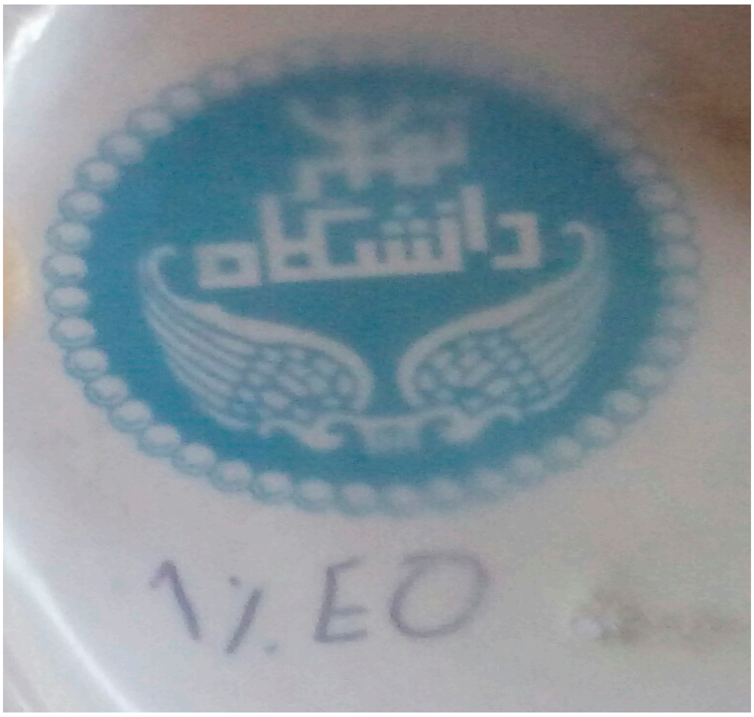

PLA+1% EO.

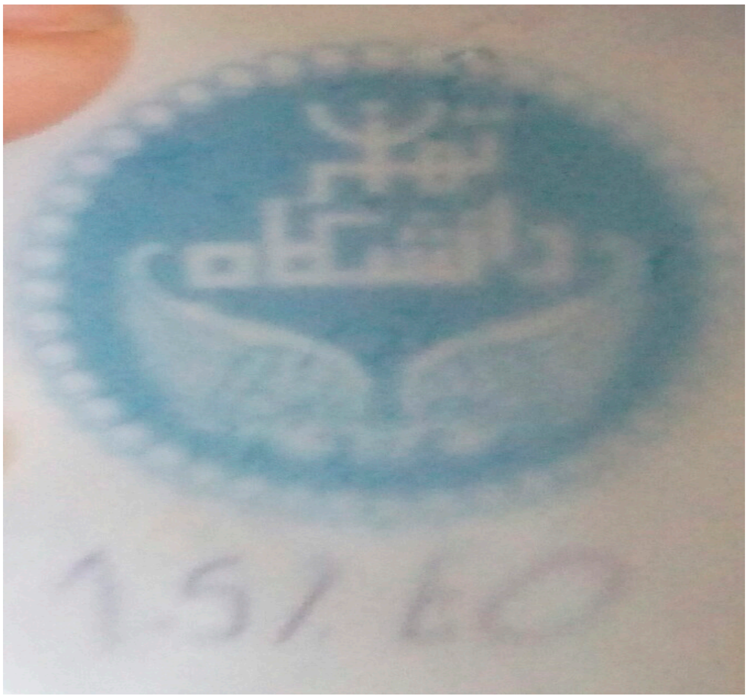

PLA+1.5% EO.
